# An Analysis of a Novel, Short-Term Therapeutic Psychoeducational Program for Children and Adolescents with Chronic Neurological Illness and Their Parents; Feasibility and Efficacy

**DOI:** 10.3389/fnins.2017.00311

**Published:** 2017-05-31

**Authors:** Bonglim Joo, Young-Mock Lee, Heung Dong Kim, Soyong Eom

**Affiliations:** ^1^Department of Pediatrics, Severance Children's Hospital, Yonsei University College of MedicineSeoul, South Korea; ^2^Department of Pediatrics, Gangnam Severance Hospital, Yonsei University College of MedicineSeoul, South Korea; ^3^Division of Pediatric Neurology, Severance Children's Hospital, Yonsei University College of MedicineSeoul, South Korea; ^4^Epilepsy Research Institute, Yonsei University College of MedicineSeoul, South Korea

**Keywords:** chronic illness, parent program, epilepsy, psychological comorbidity, quality of life, behavior

## Abstract

The purpose of this intervention was to develop a therapeutic psycho-educational program that improves quality of life in children and adolescents who are experiencing chronic neurological illness, including epilepsy, and their parents, and to analyze the intervention's feasibility and efficacy and participants' satisfaction. Participants were eight children (*n* = 8) and adolescents and their parents; participating children were experiencing chronic neurological illness with psychological comorbidity; children with intellectual impairment were excluded (IQ < 80). The program was carried out weekly for four sessions. In each of the 4 weeks, children's session content addressed self, emotion, coping skills, and finishing up, respectively; and parents' session content targeted family dynamic and emotional intervention, coping skills, childcare and education, and finishing up, respectively. Clinical psychologists administered psychological assessments (viz., Child Behavior Checklist, Pediatric Quality of Life Inventory, Parenting Stress Index, Beck Depression Inventory, Children's Depression Inventory, and Revised Children's Manifest Anxiety Scale) at pre- and post-intervention, and administered satisfaction surveys following the intervention. Participants' opinions about the program's necessity, contents, and process, and participants' overall program satisfaction were analyzed. Parents and children reported high levels of satisfaction with the program. Externalizing behavioral problems, anxiety/depression, and emotional functioning from quality of life showed improvement after the intervention. Although not statistically significant, total child stress trended downward from pre- to post-intervention. A four-session structured therapeutic psycho-educational program for children and adolescents with chronic neurological illness and their parents was successfully implemented, showing good compliance and high satisfaction and efficacy.

## Introduction

Chronic illnesses can have a significant impact on the individual and family. They are characterized by prolonged duration, failure to resolve spontaneously, and rarity of complete cure (Stanton et al., 2007); as their prevalence is estimated at < 5 to >30% in the pediatric population-in prior prevalence studies, published estimates of the proportion of children with one or more chronic illnesses range (Newacheck and Taylor, 1992). Children and adolescents with chronic illnesses face particularly significant stress and an associated risk of emotional and behavioral problems. It may affect children's life more illness-specific emotional and behavioral problems (Lavigne and Faier-Routman, 1992). Challenges and stressors resulting from chronic illnesses in childhood and adolescence are often unanticipated, uncontrollable, and functionally impairing for parents and children alike. Therefore, stress related to chronic illness affects families as well as the chronically ill children (Compas et al., 2012; Cousino and Hazen, 2013).

Regarding the family system's well-being, it is important for parents to “resolve” the child's diagnosis, a process of integrating information and emotions that results in parents adapting to the diagnosis and reorganizing caregiving behaviors (Pianta et al., 1996). Parents who resolve their child's diagnosis move on from their grief and control their distress and preoccupation with the illness' causes in order to accept the diagnosis and cope with the situation; this process naturally promotes parent–child relationships (Popp et al., 2014).

Improving psychological and physical states associated with children and adolescents' chronic illness requires psychological interventions that facilitate coping with the illness, as psychological, physical, and social factors jointly affect the illness' developmental course (Beale, 2006). Epilepsy is among the most common childhood neurological disorders; it typically involves psychological, social, and physical comorbid conditions as well as seizures, making it a heterogeneous and complex condition (Asato et al., 2009; Lewis et al., 2010). Kanner et al. has documented depression's negative effect on the control of seizure disorders: children with epilepsy are more likely to develop behavioral problems and psychiatric disorders; depression is the most frequent psychiatric comorbidity in epilepsy (Kanner et al., 2012). A review of psychological interventions targeting parents of children and adolescents with chronic illness identified the effectiveness of psychological interventions that include cognitive behavior therapy (CBT), family therapy, and multi-systemic therapy on family functioning, parent behavior and mental health, and child behavior or disability, mental health, and symptoms (Eccleston et al., 2015). Additionally, CBT was significantly more effective when parents were involved (Eccleston et al., 2009a,b). A review of psychosocial variables using a family stress framework included stressors, perceptions, adaptive resources, coping, and family adjustment (Austin and Caplan, 2007).

The purpose of the study was to develop a short-term, therapeutic psychoeducational program that addresses comorbid mental health problems and improved quality of life in children, adolescents, and their parents experience chronic neurological illness. This study also tested the developed program's feasibility and efficacy and measured participants' satisfaction following the intervention.

## Materials and methods

### Participants

Participants were recruited between June and November 2015. Candidate selection criteria were as follows: (a) children with chronic neurological illness experiencing psychosocial problems who had visited the pediatric clinic of Severance Children's Hospital in South Korea; (b) children and adolescents aged 7–18 years and their parents. The intervention was conducted at a single site with open enrollment if the participant had a chronic neurological illness. Only one exclusion criteria was that intellectually impaired children and adolescents were excluded (IQ < 80) as the intervention involved cognitive-behavioral tasks requiring verbal comprehension.

### Procedure (Table 1)

Internationally, programs developed to address pediatric chronic neurological disorders target the parent's role as well as the child's ability. Interventions addressing children and adolescents and their parents experiencing chronic neurological illness commonly aim to address a variety of problems and educational goals. Regarding children, some of these are as follows: illness behavior, stigma, adaption, social isolation, body image, quality of life (QOL), adherence, self-concept, and self-esteem. Regarding parents, some of these are as targets include: understanding one's own coping process, helping one's child manage feelings about the illness, caring for oneself, preparing one's child for communicating with others, teaching one's child coping skills, and connecting with other families. For example, children with epilepsy experience considerable psychological and psychosocial difficulties (Rodenburg et al., 2005). They will have experienced a seizure and must understand the uncertainty about when and if additional seizures will occur. They may feel that there is no control over their own lives and actions, producing additional distress. In addition, even when they are well-adjusted, the lack of stability has different possibilities and psychosocial consequences for another attack. As a result, they will have difficulty maintaining their adjustment, and struggle with issues related to interpersonal relationships, self-esteem, and having a job (Duncan, 1990). Depending on the time that epilepsy is diagnosed, the reaction of the family can influence the patient's emotional and adaptive development (Sheeran et al., 1997). When a child is diagnosed, the parents may experience fear and anxiety related to their child, as well as guilt, overprotectiveness, and mourning, which can influence the child's ability to accept their status as well as their self-concept and social adaptation (Austin et al., 1995).

**Table 1:** Therapeutic educational program.

	**Child**	**Parent**	**Time**
	**Enroll/pre-evaluation**		
Session 1	Self self-concept, self-image - stigma - body image - self esteem	Family dynamic, emotional intervention - Parenting history and review - Emotional ventilation and soothing - Managing frustration - Family dynamics	60 min
Session 2	- Emotion - managing negative emotion (anger, depression, anxiety) - emotion regulation	Coping skills - Stress management (parenting stress check, learning stress coping skills) - Mindfulness techniques	50 min
Session 3	- Coping skills - awareness of stress symptoms - learning stress coping skills, problem solving - social skills training	- Child care and education - Improving parenting efficacy - Parent–child communication training	50 min
Session 4	Finishing up - career interest planning - review and feedback collection	Finishing up - Improving family quality of life - family strength - review and feedback collection	60 min
Satisfaction surveys/post-evaluation			

In this context, this psycho-educational program was composed of four sessions administering distinct content to children and parents (Table 1). The sessions were provided by clinical psychologists. Children's session topics were as follows: session one, the self; session two, emotions; session three, coping skills; session four, finishing up. Parents' sessions were as follows: session one, family dynamics and emotional intervention (parenting history and review, emotional ventilation, and soothing, dealing with frustration, family dynamics); session two, coping skills (stress management; parenting stress check, learn stress coping skills; mindfulness technique); session three, childcare and education (improving parenting efficacy, parent–child communication training); and session four, finishing up (improving family quality of life, family strength, review of and feedback on program), which was a between 50- and 60-min session.

Additionally, psychological evaluations were administered at pre-and post-intervention to assess parents and children's adaptive and psychosocial functioning, behavioral problems, negative emotions, and quality of life; parents' depression and parenting stress were also measured. Clinical psychologists administered psychological assessments and interacted one-to-one with children and parents. At the program's close, participants discussed the program and completed comprehensive post-evaluation and satisfaction surveys.

The results of the intervention/program were then statistically analyzed. Parents provided written informed consent and the children assented as appropriate for their age. All procedures were approved by the institutional review board of Severance Hospital, Yonsei University College of Medicine.

### Assessment tools

#### Child behavior checklist (CBCL)

The Korean version of the CBCL has been validated in Korean children and adolescents (Oh and Lee, 1997). The CBCL is a parent-completed questionnaire containing 118 behavior-related statements with a standardized *t* score (mean = 50 ± 10); it includes a social competence scale and a behavior problem scale. Regarding the internalizing and externalizing scale, *t* scores > 64 (92nd percentile) were considered clinically significant; *t* scores > 70 on the behavior problem scale (98th percentile) and *t* scores lower than 36 on the competence scale (98th percentile) were considered clinically significant.

#### The Korean translation of the pediatric quality of life inventory generic core scale v.4 (PedsQL)

The Korean version of the PedsQL has been validated in Korean children and adolescents as a 23-item measure of health-related quality of life composed of four generic core scales (physical functioning, emotional functioning, social functioning, and school functioning; Varni et al., 2001). The PedsQL is a child self-report and parent proxy-report instrument that assesses the child and parent's QOL and functioning; its age ranges are 8–12 years (child version) and 13–18 years (adolescent version).

#### Children's depression inventory (CDI)

The Korean version of the CDI is a 27-item, self-administered measure of changes in depressive symptoms. Higher scores on the CDI indicate more severe depression. The CDI generates a total score and scores on three subscales: negative self-esteem, interpersonal problems, and negative mood (Kim et al., 2005).

#### Revised children's manifest anxiety scale (RCMAS)

The Korean version of the RCMAS is a 37-item self-administered questionnaire measuring anxiety in children. Higher scores on the RCMAS indicate greater anxiety. The measure comprises four subscales: worry, oversensitivity, physiological anxiety, and negative mood (Choi and Cho, 1989).

#### Beck depression index (BDI)

The Korean version of the BDI was standardized for adults as a 21-item measure of depression (Moon, 2001). In this study, mothers completed the BDI. The measures' total score is calculated by summing all item scores. Scores < 10 indicate no significant depression, 10–15 indicates mild depression, 16–23 indicates moderate depression, 24–63 indicates severe depression.

#### Parenting stress index (PSI)

The Korean version of the PSI has been standardized in Korean parents (Chung et al., 2008). The PSI generates a total stress score and scores on 13 subscales in two broad domains: stress related to characteristics of the child (the “Child Domain”) and stress related to characteristics of the parent (the “Parent Domain”). High scores in the PSI's Parent Domain suggest that dimensions of the parent's functioning may be related to stress and potential dysfunction in the parent-child system. The sum of the PSI's two domains measures total stress in the parent–child interaction; scores at or above the 85th percentile were considered clinically significant.

### Statistical analysis

Statistical analysis was performed using SPSS Statistics 20.0. Feasibility of the intervention was analyzed regarding participation; satisfaction and efficacy were analyzed using pre-post evaluation. The Wilcoxon signed-rank test was used to compare pre- and post-intervention outcome values. Descriptive statistics were calculated.

## Results

### Descriptive

Participants (*n* = 8) were aged 10.6–18.3 years (13.4 ± 2.7); they comprised three boys (37%) and five girls (63%), all with their parents. All participants were experiencing chronic neurological illness: seven had been diagnosed with pediatric epilepsy, and one had been diagnosed with chronic severe headache.

### Compliance and satisfaction with the program (Table 2)

All participants attended all four sessions of the program, showing good compliance and giving a 100% completion rate. Satisfaction surveys (five point Likert scale) were used to analyze the program's necessity, contents, and process, participants' motivation for participating in the program, and participants' overall program satisfaction. Parents and children both reported high levels of satisfaction with the program (parents: 4.5 ± 0.5; children: 4.5 ± 0.5).

**Table 2:** Parent and child satisfaction.

**Survey questions**	**Parent**	**Child**
1. How satisfied were you with the overall therapeutic educational program?	4.5 ± 0.5	4.5 ± 0.5
2. How necessary did you find the therapeutic educational program?	4.6 ± 0.5	4.1 ± 0.6
2. How satisfied were you with the quality of its contents?	4.8 ± 0.5	4.6 ± 0.5
4. Would you recommend the program to others?	4.9 ± 0.4	3.8 ± 0.9
5. Did you notice any changes in your (or your child's) thoughts or emotions over the course of the program?	4.8 ± 0.5	4.1 ± 0.8

*Numbers are the means of satisfaction survey results; responses used a 5-point Likert scale (1 = “not at all” to 5 = “very much”)*.

Parents made positive subjective comments, including the following: “It allowed me to resolve my stress by talking to others about my child” (Parent #3); “It offered practically helpful ways of doing things” (Parent #5); “Previously, I only took care of my child and didn't understand my child's thoughts or feelings or my own, but the program gave me the chance to reflect on them. In addition, I was able to understand what I should do from now on. It allowed me to understand my child better” (Parent #7); “It allowed me to learn about things I didn't know about in myself and fix my weaknesses” (Child #7).

Parents and children indicated perceiving the program as highly necessary (parents: 4.6 ± 0.5; children: 4.1 ± 0.6). Parents reported higher levels than children (parents: “very much” and “quite a lot,” 63 and 38%, respectively; children: “very much” and “quite a lot,” 25 and 63%, respectively). In addition, parents and children reported high levels of satisfaction with the quality of the program's content (parents: 4.8 ± 0.5; children: 4.6 ± 0.5); particularly, parents reported no dissatisfaction with program content (“not at all,” “a little”: #0%, 0%, respectively.).

Parents and children made the following comments: “It was satisfying to see myself as a mother and learn about specific ways I can increase my strength in coping with stressful situations. I was also very satisfied with the specific strategies the program provided, which I can directly apply to my child” (Parent #5); “It was very satisfying to learn about ways to relieve stress, as they allow me to resolve stress without troubling others” (Child #8).

Regarding the program's process, the administrators' attitude was rated as very satisfactory (parents, “very much”: 100%; children, “very much”: 100%). Most importantly, parents and children both reported noticing positive changes in their child's (or their own) thoughts or emotions (parents: 4.8 ± 0.5; children: 4.1 ± 0.8). Related comments were as follows: “I stopped blaming myself and tried to look at the situation objectively, and the program gave me motivation to overcome difficulties” (Parent #5); “I was too depressed about my child's illness, but after the program, I felt the need to take care of things other than the disease itself” (Parent #6); “My feelings and thoughts became more positive” (Child #3); “As I learned about myself, I was able to start conversations with my friends and become closer to them” (Child #7).

Participants also indicated wishing to recommend the program to others (parents: 4.9 ± 0.4; children: 3.8 ± 0.9): “The program gives direction to people who are experiencing psychological difficulties, so I hope many people can experience this program” (Parent #5); “I like programs for the child, but I wish there were more programs for parents. It is important to comfort the parents' feelings, but I would like to learn more about how to take care of my sick child. Physical caring is good, but there are also a lot of parents who do not know how to take care of themselves mentally” (Parent #6); “It was a good time to think about myself more by meeting four times for the past 4 weeks” (Child #7).

### The intervention's effects on behavioral problems (Table 3)

Regarding behavioral problem scores on the CBCL, particularly, externalizing behavior problems decreased significantly (62.1 ± 4.7 vs. 57.7 ± 9.5, *p* < 0.05). Regarding the CBCL's subdomains, scores on “anxiety/depression” and “other” decreased significantly (64.4 ± 10.9 vs. 60.2 ± 10.5, *p* < 0.05; 63.5 ± 6.9 vs. 59.2 ± 8.1, *p* < 0.05, respectively).

**Table 3:** Competence and behavioral problems resulting from K-CBCL at pre and post-intervention (*N* = 8).

**CBCL variables**	**Pre-intervention**	**Post-intervention**	***p*^a^**
**COMPETENCE**			
Total Competence	41.5 (14.2)	41.3 (8.7)	0.478
Social Competence	39.1 (10.0)	38.6 (7.8)	0.725
School Competence	48.6 (12.4)	48.5 (9.7)	0.893
**BEHAVIORAL PROBLEMS**			
Total behavior problems	69.5 (8.8)	65.2 (10.3)	0.119
Internalizing problems	70.6 (13.5)	66.7 (15.5)	0.079
Externalizing problems	62.1 (4.7)	57.7 (9.5)	0.028^*^
Anxiety/Depression	64.4 (10.9)	60.2 (10.5)	0.041^*^
Anxious/Withdrawn	67.6 (7.5)	64.2 (9.4)	0.233
Somatization	64.0 (9.1)	63.2 (10.2)	0.588
Social problems	69.4 (8.3)	67.8 (9.5)	0.248
Thought problems	62.1 (7.3)	61.7 (7.8)	0.891
Attention problems	63.0 (9.7)	58.2 (5.9)	0.092
Rule breaking	60.0 (6.3)	58.2 (5.9)	0.400
Aggressive behavior	61.0 (5.7)	58.6 (6.9)	0.176
Other	63.5 (6.9)	59.2 (8.1)	0.011^*^
**DSM DIAGNOSTIC CRITERIA**			
Emotional problems	65.1 (9.9)	63.1 (8.4)	0.235
Anxiety problems	64.8 (12.3)	60.6 (10.6)	0.078
Somatization	61.1 (10.1)	61.3 (9.1)	0.785
ADHD	57.0 (4.9)	55.6 (5.3)	0.268
Oppositional Defiant problems	58.6 (6.9)	57.1 (7.5)	0.400
Conduct problems	59.6 (5.6)	59.0 (5.9)	0.786

p^a^, p-value by non-parametric Wilcoxon signed-rank test comparing pre-intervention and post-intervention;

* *p < 0.05. All scores are age-adjusted t-scores with mean = 50, SD = 10. K-CBCL, Korea-Child Behavior Checklist; DSM, Diagnostic and Statistical Manual of Mental Disorders; ADHD, attention deficit hyperactivity disorder*.

However, the limited statistical significance was produced by the intervention due to the low number of participants; Total behavioral and internalizing behavior problem scores trended downward from pre- to post-test (69.5 ± 8.8 vs. 65.2 ± 10.3, *p* = 0.119; 70.6 ± 13.5 vs. 66.7 ± 15.5, *p* = 0.079, respectively). Scores on the following subdomains decreased without reaching statistical significance: anxiety/withdrawal (67.6 ± 7.5 vs. 64.2 ± 9.4, *p* = 0.233), social problems (69.4 ± 8.3 vs. 67.8 ± 10.2, *p* = 0.248), attention problems (60.0 ± 9.7 vs. 58.2 ± 5.9, *p* = 0.092), rule-breaking (60.0 ± 6.3 vs. 58.2 ± 5.9, *p* = 0.400), and aggressive behavior (61.0 ± 5.7 vs. 58.6 ± 6.9, *p* = 0.176). Competence scores showed no significant differences following the intervention, as well.

### The intervention's effect on quality of life (Table 4)

Among the parent-report on child's QOL, emotional functioning showed significant improvement (66.9 ± 15.1, *p* < 0.05).

**Table 4:** Effect of intervention on QOL (*N* = 8).

	**Child**			**Parent**		
	**Pre-intervention**	**Post-intervention**	***p*^a^**	**Pre-intervention**	**Post-intervention**	***p*^a^**
Physical functioning	62.8 (16.2)	64.1 (13.9)	0.779	70.7 (21.3)	71.3 (16.4)	0.866
Emotional functioning	58.1 (24.0)	62.5 (18.3)	0.308	57.5 (16.6)	66.9 (15.1)	0.040^*^
Social functioning	63.7 (30.5)	58.8 (28.6)	0.518	67.5 (34.5)	76.3 (22.1)	0.463
School functioning	59.3 (20.2)	63.8 (21.0)	0.574	62.5 (23.4)	65.0 (24.0)	0.734

*p^a^, p-value by non-parametric Wilcoxon signed-rank test comparing pre-intervention and post-intervention. QOL, quality of life*.

Children's self-reported QOL showed no significant differences from pre- to post-intervention; however, scores for emotional functioning and school functioning improved (58.1 ± 24.0 vs. 62.5 ± 18.3, *p* = 0.308; 59.3 ± 20.2 vs. 63.8 ± 21.0, *p* = 0.574, respectively). Additionally, social functioning and school functioning trended toward improvement from pre-intervention but failed to reach statistical significance (67.5 ± 34.5 vs. 76.3 ± 22.1, *p* = 0.463; 62.5 ± 23.4 vs. 65.0 ± 23.0, *p* = 0.734).

### The intervention's effect on parenting stress (Supplementary Table 1)

At the parenting stress baseline, reinforcement, demandingness, acceptability, child total stress, competence, attachment, health, and total parent stress were considered at clinically significant levels (scores at or above the 85th percentile). Regarding the K-PSI, significant differences were not seen from pre- to post-intervention in total parenting stress scores (Child Domain and Parent Domain); however, child total stress decreased in the Child Domain. Particularly, subscales including distractibility/hyperactivity and demandingness decreased but failed to reach statistical significance (42.5 ± 31.2 vs. 36.2 ± 28.5, *p* = 0.340; 93.1 ± 4.7 vs. 87.5 ± 10.0, *p* = 0.204, respectively). In the Parent Domain, attachment and role restriction trended downward (89.5 ± 12.0 vs. 87.8 ± 12.7, *p* = 0.441; 59.1 ± 34.2 vs. 54.5 ± 31.7, *p* = 0.136, respectively).

### The intervention's effect on negative emotions (Supplementary Table 2)

Children and adolescents' negative emotion scores showed no significant differences after intervention; however, depression scores decreased non-significantly (12.1 ± 6.3 vs. 10.1 ± 7.1, *p* = 0.236). In addition, mothers' depression trended downward from pre-intervention but failed to reach statistical significance (18.9 ± 12.6 vs. 15.8 ± 9.0, *p* = 0.360).

## Discussion

### Feasibility and efficacy of the intervention

This study's results support the efficacy of the developed therapeutic educational program for chronic neurological illness. Parents of children with a chronic illness experience ongoing stressors; hence, educational interventions naturally significantly improve parents' mental health (Kyngäs and Rissanen, 2001; Jerram et al., 2005; El-Mallakh et al., 2010; Davis and Conroy, 2015). Previous studies have noted the need for educational interventions in families (May and Pfäfflin, 2005); education programs for patients are therefore now part of chronic disease management and provide participants with confidence, self-efficacy, and the belief that the disease is under their control.

Parents and children both reported high levels of satisfaction with the psychological support provided throughout the program. Parents' survey responses indicated higher levels of satisfaction than children's did; this may reflect several psychological considerations. Centrally, our program included children and their parents; moreover, both sets of participants confirmed the program's feasibility and necessity. Session content targeting children addressed negative self-concept, low self-esteem, stigma (particularly regarding body image), adaptation, emotion, and quality of life. This session content was included as earlier research indicates that targeted interventions may decrease stigma and other psychological comorbidities in individuals with epilepsy (Hauser, 1994; MacLeod and Austin, 2003), which individuals diagnosed with epilepsy commonly experience. In contrast, session content targeting parents addressed emotion management, coping skills, and communicating with their children. The difference between children's and parents' session content may thus explain children's and parents' differing levels of satisfaction.

Satisfaction survey results indicated that the program importantly addressed participants' emotional wellness as well as the illness itself. The program is therefore well-suited for implementation in addressing topics common in chronic illness, as it provided practical information and strategies to successfully manage challenges facing children and their parents experiencing neurological disorder. The program's administrators primarily focused on encouraging participation in the program, as well as encouraging emotional exchange between children and their parents. Consequently, the program improved parents' psychological health and provided them with additional social support, corroborating earlier research (Distelberg et al., 2014). In addition, the administrators focused on practical strategies in one-to-one interventions; further, parents were encouraged to develop personalized coping strategies. These considerations may explain the program's high satisfaction ratings.

The results of this study do not support statistical significance or clinical impact, given the low number of subjects and lack of power analysis. Therefore, the current results do not support the statement that the program should be deployed in additional clinical settings, but rather that the pilot program demonstrated strong satisfaction scores and that a larger, and more controlled study would be needed to determine overall clinical impact and generalizability. Furthermore, despite such limitations, this study provides initial evidence supporting the develop program as a potentially effective method to provide effective and integrated therapeutic intervention promoting the mental health of children and adolescents and their parents experiencing chronic neurological illness, in addition to treating this illness itself; this program may be implemented in healthcare departments addressing pediatric diseases. Additionally, it may be feasible to deploy the program in a clinical setting by compiling guided materials for the use of clinicians such as physicians and therapists.

### Psychological effects of the intervention

Epilepsy can seriously impair quality of life in children and their families. This population has much higher rates of psychological, social, and academic difficulties than youth with other chronic health conditions or youth in general. Depression, parenting stress, and the child's overall quality of life are critical to effectively managing psychological comorbidity in children with epilepsy (Cushner-Weinstein et al., 2008). Parents of children with epilepsy are at high risk of anxiety; additionally, parental anxiety is significantly related to children's QOL (Li et al., 2008). Psycho-educational programs such as the SEE (Shore et al., 2008), FLIP&FLAP (Jantzen et al., 2009), and FAMOSES (Pfäfflin et al., 2012) target children with epilepsy and their parents, effectively reducing parental anxiety and improving QOL in children and their parents. The program deployed in this study measurably improved scores on indices of psychological functioning; particularly, positive changes were observed following the program in children's externalizing behavior problems, anxiety/depression, and emotional functioning, despite the program's comparatively short duration. This may be because externalizing behavior is partly positively affected by parenting practices and parental emotional support (Willemen et al., 2011); however, we found no significant change in parents' competence and children's QOL after the program, despite substantial levels of participant satisfaction. Significant changes in variables such as competence or QOL may thus require greater program duration to manifest (Eom et al., 2014).

### Limitations and future directions

Our results support the feasibility and effectiveness of the present program; however, our study has some limitations. We did not perform a controlled prospective study; no control group was used. Additionally, the intervention was performed at a single site and the number of participants was small, which limited the author's ability to detect overall strength of the program: only eight children and their parents participated. Further, we limited participants to individuals experiencing chronic neurological illness, and most participating children had epilepsy. These considerations limit our findings' generalizability. Finally, only children with age-appropriate cognitive abilities may participate in the program; this limits our findings' generalizability and the program's potential range of application.

Future research should aim to deploy similar programs in larger samples including a variety of disorders, and should measure and analyze additional illness-specific variables (e.g., type of epilepsy, age of onset, duration of illness). Future research should also develop programs able to address subgroups in the pediatric patient population, such as patients with intellectual disabilities and inpatients. Deploying programs of various durations would allow time-efficient intervention tailored to particular morbidities and permit examination of program efficacy as a function of duration. Finally, research should identify means of preventing participant relapse following program cessation (Eccleston et al., 2015).

## Conclusion

Demonstrating the support of a pilot, short-term psychotherapy intervention that demonstrated good patient satisfaction and may represent a feasible intervention that could be effectively utilized secondary to minimal length and relatively limited resources required to complete the intervention. Moreover, clinical psychologists could be a part of the multidisciplinary team treating neurological disorders providing the psychoeducational intervention. However, the data presented do not support implementation of the program as minimal statistically significant results were found in the outcome measures outside of patient/parent satisfaction and no clinical statistics (affect size or number needed to treat) were completed.

## Author contributions

BJ and SE were responsible the conception and organization of the research project, and contributed to drafting and revising the work. SE contributed to revising the work and to final approval of the version as a corresponding author. BJ and SE were responsible for the design and execution of statistical analysis and interpretation of data for the analysis. YL and HK were responsible for the review of clinical variables and revising the work. All authors reviewed and revised the manuscript, approved the final manuscript as submitted, and have approved the acknowledgment of their contributions.

### Conflict of interest statement

The authors declare that the research was conducted in the absence of any commercial or financial relationships that could be construed as a potential conflict of interest. The reviewer EB and handling Editor declared their shared affiliation, and the handling Editor states that the process nevertheless met the standards of a fair and objective review.
