# Supplementary material for: An Analysis of a Novel, Short-Term Therapeutic Psychoeducational Program for Children and Adolescents with Chronic Neurological Illness and Their Parents; Feasibility and Efficacy
Source: Front Neurosci. 2017 May 31;11:311. doi: 10.3389/fnins.2017.00311 (PMC5450004; doi:10.3389/fnins.2017.00311)
Supplement: Supplementary file 1 [file Table1.PDF]

Supplementary Table 1. Parenting stress from K-PSI and at pre- and post-intervention (N = 8)

	Variables	Pre-intervention	Post-intervention	p^a
PSI	Total Parenting Stress	90.9 (11.8)	92.3 (7.9)	0.655
	Total Child Stress	88.6 (13.2)	87.8 (11.3)	0.833
	Distractibility/Hyperactivity	42.5 (31.2)	36.2 (28.5)	0.340
	Adaptability	78.1 (31.8)	78.0 (30.1)	0.350
	Reinforcement	93.0 (5.2)	93.8 ((5.4)	0.726
	Demandingness	93.1 (4.7)	87.5 (10.0)	0.204
	Mood	57.9 (33.0)	69.3 (27.9)	0.075
	Acceptability	89.5 (24.0)	95.7 (5.3)	0.854
	Total Parent Stress	88.9 (17.7)	92.8 (7.9)	0.998
	Competence	87.5 (15.7)	90.2 (11.2)	0.306
	Isolation	73.3 (38.1)	77.1 (27.6)	0.500
	Attachment	89.5 (12.0)	87.8 (12.7)	0.441
	Health	81.0 (12.7)	82.1 (11.0)	0.865
	Role Restriction	59.1 (34.2)	54.5 (31.7)	0.136
	Depression	78.4 (31.9)	82.2 (25.6)	0.674
	Spouse	67.5 (28.5)	69.6 (17.0)	0.611

p^a, *p*-value by non-parametric Wilcoxon signed rank test comparing pre-intervention and post-intervention in SPSS; *$p < .05$. PSI scores are age-adjusted percentile scores; K-PSI, Korea-Parenting Stress Index.

Supplementary Table 2. Impact of intervention on negative emotions (N = 8)

	Pre-intervention	Post-intervention	p[a]
Child			
Depression (CDI)	12.1 (6.3)	10.1 (7.1)	0.236
Anxiety (RCMAS)	10.9 (5.0)	11.0 (4.2)	0.975
Parent			
Depression (BDI)	18.9 (12.6)	15.8 (9.0)	0.360

p[a], *p*-value by non-parametric Wilcoxon signed rank test comparing pre-intervention and post-intervention in SPSS; *$p < .05$. CDI, Children's Depression Inventory; RCMAS, Revised Children Manifest Anxiety Scale; BDI, Beck Depression Index.
